# Cyto-Genotoxicity of Tritiated Stainless Steel and Cement Particles in Human Lung Cell Models

**DOI:** 10.3390/ijms231810398

**Published:** 2022-09-08

**Authors:** Yordenca Lamartiniere, Danielle Slomberg, Michaël Payet, Virginie Tassistro, Alice Mentana, Giorgio Baiocco, Jerome Rose, Laurence Lebaron-Jacobs, Christian Grisolia, Véronique Malard, Thierry Orsière

**Affiliations:** 1 Aix Marseille Univ, Avignon Univ, CNRS, IRD, IMBE, F-13005 Marseille, France; 2 Aix-Marseille Univ, CNRS, IRD, INRAE, Coll. De France, CEREGE, 13545 Aix-en-Provence, France; 3 CEA, IRFM, 13108 Saint Paul lez Durance, France; 4 Laboratory of Radiation Biophysics and Radiobiology, Department of Physics, University of Pavia, 27100 Pavia, Italy; 5 Aix Marseille Univ, CEA, CNRS, BIAM, IPM, F-13108 Saint Paul-Lez-Durance, France

**Keywords:** stainless steel particles, cement particles, tritium, in vitro testing, cytotoxicity, micronuclei, DNA damage, chromosome damage, BEAS-2B cells, MucilAir^TM^

## Abstract

During the decommissioning of nuclear facilities, the tritiated materials must be removed. These operations generate tritiated steel and cement particles that could be accidentally inhaled by workers. Thus, the consequences of human exposure by inhalation to these particles in terms of radiotoxicology were investigated. Their cyto-genotoxicity was studied using two human lung models: the BEAS-2B cell line and the 3D MucilAir^TM^ model. Exposures of the BEAS-2B cell line to particles (2 and 24 h) did not induce significant cytotoxicity. Nevertheless, DNA damage occurred upon exposure to tritiated and non-tritiated particles, as observed by alkaline comet assay. Tritiated particles only induced cytostasis; however, both induced a significant increase in centromere negative micronuclei. Particles were also assessed for their effects on epithelial integrity and metabolic activity using the MucilAir^TM^ model in a 14-day kinetic mode. No effect was noted. Tritium transfer through the epithelium was observed without intracellular accumulation. Overall, tritiated and non-tritiated stainless steel and cement particles were associated with moderate toxicity. However, these particles induce DNA lesions and chromosome breakage to which tritium seems to contribute. These data should help in a better management of the risk related to the inhalation of these types of particles.

## 1. Introduction

Tritium is a radioactive isotope of hydrogen, with a physical half-life of 12.3 years. It is a beta emitter since its decay leads to the emission of an electron with a mean energy of 5.7 keV. The main natural source of tritium results from the action of cosmic rays on the atmosphere, and the main anthropogenic source of tritium is associated with the nuclear industry [1]. The beta particles emitted by the radioactive decay of tritium are not energetic enough to penetrate the outer layer of the human skin. Therefore, tritium poses a human health risk only after internal exposure through ingestion, inhalation, and absorption through the skin [1,2]. Ingestion mostly contributes to the exposure of the general population while inhalation and absorption are the main routes of intake at the workplace. As an isotope of hydrogen, tritium forms HTO molecules that behave like water in the body. Once in the body, the biological half-life of tritium is estimated to be about 10 days, as HTO is incorporated into the body and eliminated relatively quickly, like H_2_O [3]. A fraction of tritium may also be incorporated into organic molecules, forming organically bound tritium (OBT). OBT can remain in the body for longer periods of time, with two biological half-lives of about 40 days and about a year, depending on the turnover of organic molecules that have incorporated the tritium [3]. Following OBT formation, the elimination of tritium could be slowed down, resulting in a higher absorbed radiation dose and more severe cell damage.

Tritium is the main radionuclide released by current nuclear power plants at an estimated level of 0.1 EBq per year [1] Due to the growth of nuclear power generation worldwide and to the development of nuclear fusion technology, the risk of tritium release into the environment is increasing. During the decommissioning of nuclear facilities, operations are intended to remove or eliminate any tritiated material. Tritium interacts with and easily permeates into building materials, and during decommissioning operations micrometric and sub-micrometric tritiated stainless steel and cement particles are mainly generated. Such particles are respirable and could deposit in various compartments of the respiratory tract. In case of accidental exposure during dismantling, workers may inhale particles and this could constitute a health risk for which there is currently a lack of information. In the framework of the TRANSAT project (http://transat-h2020.eu (accessed on 25 July 2022), investigations were proposed to improve knowledge in the field of dosimetry, radiotoxicology, genotoxicology, ecotoxicology and environmental fate in case of contamination by tritiated stainless steel and cement particles [4].

Airborne particles can be inhaled and, depending on their dimensions, deposited in the various units of the respiratory tract. In case of inhalation at the workplace, tritiated particles can remain in the lungs and tissues can be exposed to both particle-related stress and beta radiation for a long time if tritium remains bound to insoluble particles. Although insoluble particles are largely retained in lung tissues, transported by macrophages to regional lymph nodes, or escalated from the lungs by mucociliary clearance, some dissolution occurs and a proportion of their tritium content is removed and absorbed as HTO [1]. Cytogenetic damage was analyzed in mice chronically exposed to HTO and the genotoxic potential of low doses of tritium (>10 kBq/L) was shown to be higher than currently assumed [5]. Moreover, exposure to particles could itself induce injury following inhalation. Indeed, construction workers exposed to cement particles (mainly by inhalation) can carry enhanced nuclear aberrations in their lymphocytes or epithelial buccal cells [6,7]. In contrast, stainless steel particle exposure by inhalation was associated with very low toxicity in vitro and in vivo [8].

Recently, several studies evaluated the cyto-genotoxicity of tritiated tungsten particles that could be generated in nuclear fusion facilities (ITER-type reactor). More severe cytogenetic effects were reported in the bronchial human-derived epithelial BEAS-2B cell line following in vitro exposure to tritiated tungsten particles compared to untritiated ones [9]. In vitro studies indicated that tritium is released as HTO into simulated lung fluids from various metallic tritiated particles such as lanthanum nickel aluminum alloy, zirconium, carbon, and hafnium [10,11,12,13]. It is known that for inhalation of inorganic particulate materials, the biokinetics of tritium absorbed into body fluids follows that of HTO [14].

DNA and chromosomes are interesting targets of particulate and radiative stress as genetic damages play key roles in the pathological consequences of ionizing radiation as well as particles exposure [15]. To gain insights on damage-inducing mechanisms related to the inhalation of such particles, in vitro toxicological studies using human lung models could be useful and allow animal experimentations to be refined, reduced, and replaced. Indeed, there is a clear need to circumvent time-consuming, expensive, and ethically questionable in vivo studies. The in vitro cytokinesis-block micronucleus (CBMN) and comet assays have been widely used as simple, rapid, and sensitive tools for assessment of DNA damage and chromosome aberrations, respectively. The advantages of the comet assay are its ability to detect DNA damage in any cells, despite having non-proliferating cells, its sensitivity, and that the OECD has adopted a guideline for the in vivo comet assay. The in vitro comet assay conducted in cultured cells is commonly used for assessing the ability of various agents to induce primary DNA lesions at very low concentrations [16]. The in vitro CBMN assay is a well-established technique widely used in genetic toxicology as well as in biomonitoring for assessing genetic damage after a radiation accident or event [17,18,19]. The in vitro CBMN assay quantifies the frequency of micronuclei (MN) in binucleated cells (BNCs) in various cell lines in proliferation and is validated by the OEDC [20] (OECD TG487) to efficiently reveal both clastogenic and aneugenic agents. Micronuclei are formed from whole chromosomes or chromosome fragments that lag behind during the metaphase–anaphase transition and are not included inside one of the two main nuclei after karyokinesis.

To assess potential health effects following exposure via inhalation, these genetic toxicology tests were widely applied on the bronchial human-derived epithelial BEAS-2B cell line [9,21,22,23]. Rapid developments in the 3D culture of primary epithelial cells, the use of induced pluripotent stem cells for generation of lung epithelial cells, and the development of organ-on-a-chip technology have led to significant advances in inhalation toxicology [24]. For example, human-derived organotypic 3D airway models consisting of a fully differentiated respiratory epithelia display metabolic activity, mucus production, and cilia beating, thus allowing air–liquid exposure close to in vivo conditions. They are useful when endpoints do not need active cell proliferation [25,26,27]. The assessment of combinations of various endpoints such as epithelium integrity can be conducted. Recently, using the MucilAir^TM^ model, a 3D in vitro cell model of the human airway epithelium, a small and persisting (over seven days) effect on epithelial integrity and inflammatory response without any decrease in cell viability was documented following high-level exposure to HTO (330 kBq/well corresponding to 44 cGy/well) [28].

In this study, we used the BEAS-2B cell line and the 3D MucilAir^TM^ model to investigate the cyto-genotoxicity of tritiated stainless steel and cement particles mimicking the particles emitted during the decommissioning process of nuclear fission facilities. In order to discriminate between the tritium radiative effect and the particulate effect, hydrogenated cement and stainless steel particles were used as a control. Cell viability was evaluated via the quantification of the adenosine triphosphate (ATP) in BEAS-2B cells. Genotoxic potential of the particles was evaluated using the comet assay and the cytome version of the CBMN assay. Pancentromeric staining was performed to discriminate chromosome breakage and loss, the two mechanisms associated with micronuclei formation. Oxidative stress, which has been proposed as the factor triggering the cyto-genotoxicity of particles, has been investigated based on the ratio of the oxidized/reduced glutathione cellular content. Using the Mucilair^TM^ model, the effect of tritiated particles on barrier integrity and metabolic activity were monitored. We also analyzed the transfer of tritium across the pulmonary epithelial barrier. To our knowledge, this is the first study aiming to address the cyto-genotoxic potentials at the pulmonary level of tritiated inhalable dusts that could be generated during dismantling of nuclear facilities.

## 2. Results

### 2.1. Cyto-Genotoxicity of Tritiated Stainless Steel and Cement Particles in BEAS-2B Cells

#### 2.1.1. Behavior of Particles in Cell Culture Medium

**Figure 1:**
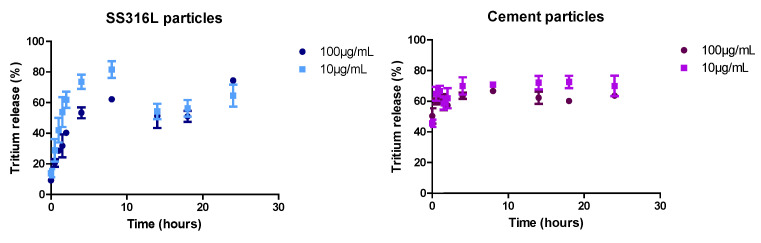
Quantification of tritium release from stainless steel and cement particles in cell culture medium. Tritiated particles were suspended in cell culture medium and incubated at 37 °C under agitation. Samples were collected at several time points and then tritium amount was assessed in the supernatant and in the particle suspension using liquid scintillation counting. Data are presented as mean ± SEM of three independent experiments.

The SS316L particles were considered insoluble at 37 °C with agitation (i.e., minimal release of Fe, Cr, and Ni) in culture medium [29], but 60% of tritium was released from the tritiated particles after 2 h of incubation, probably as HTO (Figure 1). In the culture medium, the SS316L particles exhibited an initial size distribution of ~3–6 μm (Supporting Information Figure S1). However, the SS316L particles were not stable in suspension and aggregates of ~16 µm formed within 10 min (Supporting Information Figure S2). Due to the high density (d = 7.87) of the stainless steel and the presence of these aggregates, the SS316L particles rapidly settled out of suspension. Indeed, based on Stokes law, ~3 μm SS316L particles would sediment at a velocity of 0.14 mm/s [28].

The cement particles were partially soluble, with ~60% of the Ca released into the culture medium very quickly [29]. The tritium release from the particle was around 70% after 30 minutes of incubation (Figure 1). Thus, the majority of the tritium was likely linked with Ca-containing phases in the cement. In the cell culture medium (37 °C with agitation), the 1–10 μm cement particle size distribution remained stable (Supporting Information Figure S3), and the overall particle structure was conserved.

#### 2.1.2. Observation of Particles in Cell Cultures

**Figure 2:**
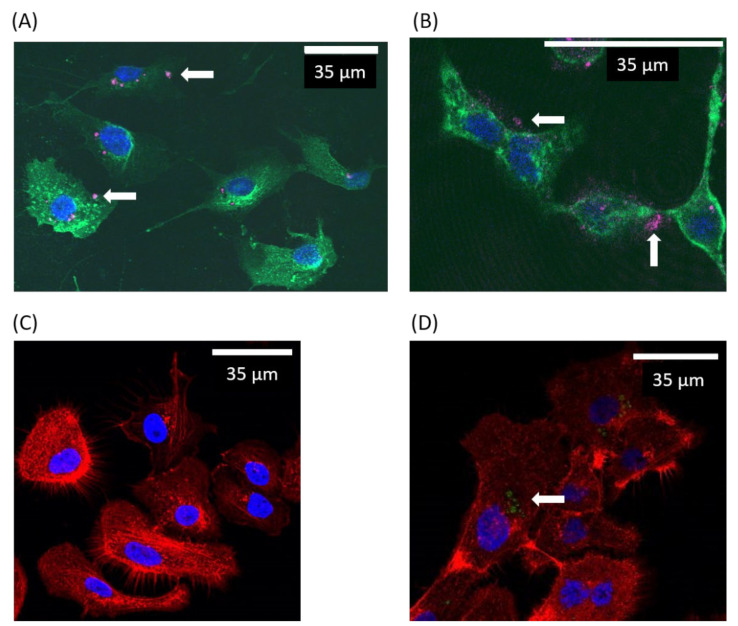
Confocal microscopy observation of BEAS-2B cells after exposure to hydrogenated particles. (**A**,**B**) After exposure of BEAS-2B cells to stainless steel (**A**) or cement (**B**) particles, staining of plasma membrane (green), nucleus (blue), and cytoskeleton (red) was performed. Images were acquired using confocal microscopy. Particles, visualized by light reflection (pink), are indicated by white arrows. (**C**,**D**) Staining of nucleus (blue) and cytoskeleton (red) was performed on untreated cells and cells exposed to stainless steel particles. The SS316L particles (green and indicated by white arrow) seem to form depressions in the cells.

After exposure of cells to SS316L particles, some SS316L aggregates of different sizes were observed (Figure 2A). As previously described, these aggregates likely formed when the SS316L particles were suspended in the aqueous media [29]. Confocal microscopy pictures showed that the aggregates were often found in the vicinity of the cell nucleus (Figure 2B), but internalization of particles by the cells was not observed.

Some depressions of the cell surface were also detected (Figure 2D). These adaptations of the cell morphology were probably associated with the high density of the stainless steel particles.

The cement particles behaved differently than the stainless steel ones. They were evenly distributed on the cell surface (Figure 2B). The particles seemed to adhere to the plasma membrane. No morphological changes of cells were observed in the presence of particles.

#### 2.1.3. Cytotoxic Effects of Tritiated Particles

Cytotoxicity, based on cellular ATP quantification, was investigated after exposure of BEAS-2B human lung cells to 0 to 200 μg/mL of hydrogenated and tritiated particles.

**Figure 3:**
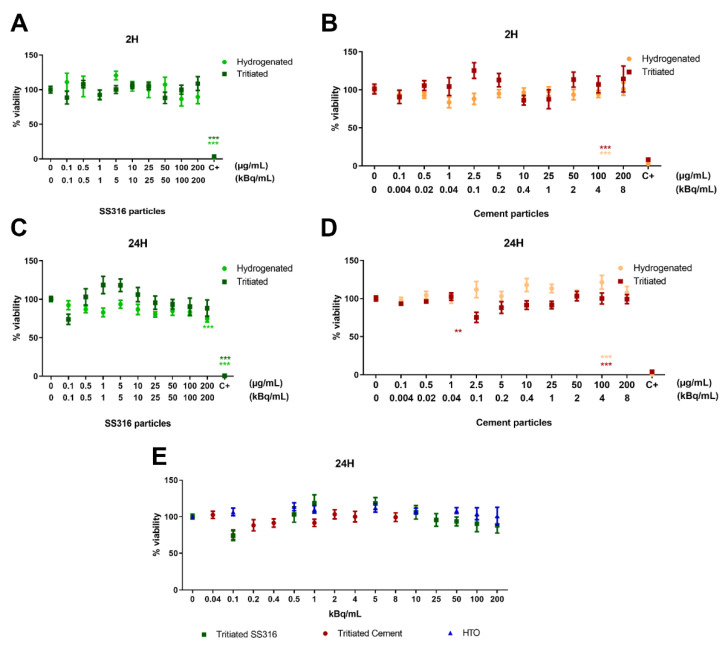
Cytotoxicity of hydrogenated and tritiated particles measured in BEAS-2B cells: (**A**) SS316L particles, 2h exposure; (**B**) SS316L particles, 24h exposure; (**C**) cement particles, 2h exposure; (**D**) cement particles, 24h exposure; (**E**) HTO, SS316L and cement particles, 24h exposure. As a positive control, cells were exposed to 9% Triton-X 100 diluted in culture medium. Data are presented as mean ± SEM of three independent experiments, each in triplicate. Statistical significance was evaluated by one-way ANOVA followed by Dunnett’s multiple comparisons test; ** *p* < 0.01, *** *p* < 0.001.

After 2 h exposure to hydrogenated and tritiated SS316L particles, none of the tested conditions showed any cytotoxic effect (Figure 3A). Following 24 h exposure, no toxicity was noted, excepted for the highest tested concentration of hydrogenated SS316L particles (200 µg/mL) (Figure 3B).

Following exposure to hydrogenated and tritiated cement particles no cytotoxic effects were detected either at 2 h or at 24 h exposure duration (Figure 3C,D).

Figure 3E shows that the viability was unchanged when cells were exposed to activities between 0 and 200 kBq/mL via tritiated particles or tritiated water for 24 h. These results suggest an absence of mitochondrial toxicity.

These cytotoxicity data allowed us to select concentrations to be tested for genotoxicity experiments. We chose to eliminate the highest concentration of stainless steel particles as a moderate toxicity was noted following 24-hour exposure.

#### 2.1.4. Genotoxic Effects Exerted by Tritiated Particles: DNA Strand Break

**Figure 4:**
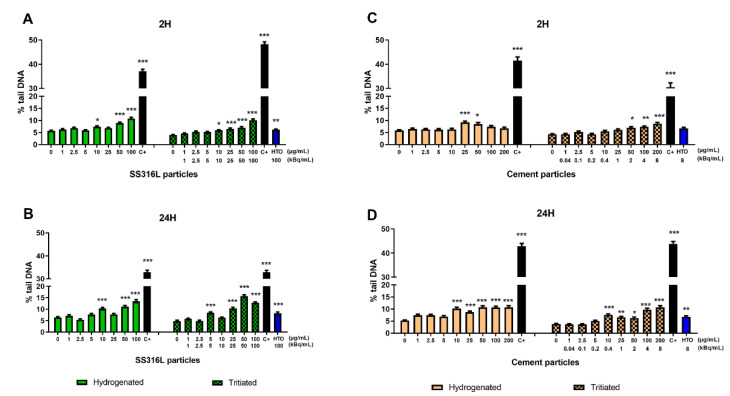
Evaluation of DNA damage induced by SS316L and cement particles, by alkaline comet assay. Percentage of tail DNA was quantified after 2 h and 24 h of exposure to hydrogenated and tritiated SS316L (**A**,**B**) or cement particles (**C**,**D**). As a positive control, cells were exposed to 110 µM hydrogen peroxide (black bars). Cells were also exposed to tritiated water with an activity corresponding to the activity of the highest tested concentration of particles (100 kBq/mL and 8 kBq/mL). Each bar represents the mean ± SEM of two to three independent experiments. Asterisks indicate statistically significant increase compared to untreated cells with *p*-value of *p* < 0.05 (*), *p* < 0.01 (**), or *p* < 0.001 (***).

The alkaline version of the comet assay, measuring mainly DNA single strand breaks and alkaline labile sites, was used to assess particle-induced DNA damage in BEAS-2B cells. As shown in Figure 4, both types of particles induce DNA single-strand breaks compared to untreated cells.

Following 2 h exposure, a statistically significant increased amount of DNA damage was seen in cells exposed to hydrogenated SS316L at concentrations of 10, 50, and 100 µg/mL and at concentrations ≥10 µg/mL with the tritiated ones (Figure 4A). The increases in primary DNA lesions induced by hydrogenated and tritiated stainless steel particles were similar following 24 h exposure (Figure 4B).

Following 2 h exposure, a statistically significant increased amount of DNA damage was seen in cells exposed to hydrogenated cement particles at concentrations of 25 and 50 µg/mL and at concentrations ≥50 µg/mL with the tritiated ones (Figure 4C).

Following 24 h exposure, cement particles induced a significant increase in DNA single strand breaks at concentrations ≥10 µg/mL. No difference in the induction of DNA damage was noted between hydrogenated and tritiated cement particles (Figure 4D).

Short and long exposure duration generated a similar amount of DNA breaks.

Tritium, in the form of HTO, was able to induce an increase in DNA breaks in BEAS-2B cells, whatever the activity (100 kBq/mL and 8 kBq/mL). The increases in primary DNA lesions induced by hydrogenated particles were so marked that we can neither exclude nor conclude that additional DNA single strand breaks due to tritium would be evidenced in these experiments. Thus, the presence of tritium could contribute to the DNA damage effect exerted by tritiated cement and SS316L particles.

#### 2.1.5. Chromosomal Damage Exerted by Tritiated Particles: Micronuclei Formation

**Figure 5:**
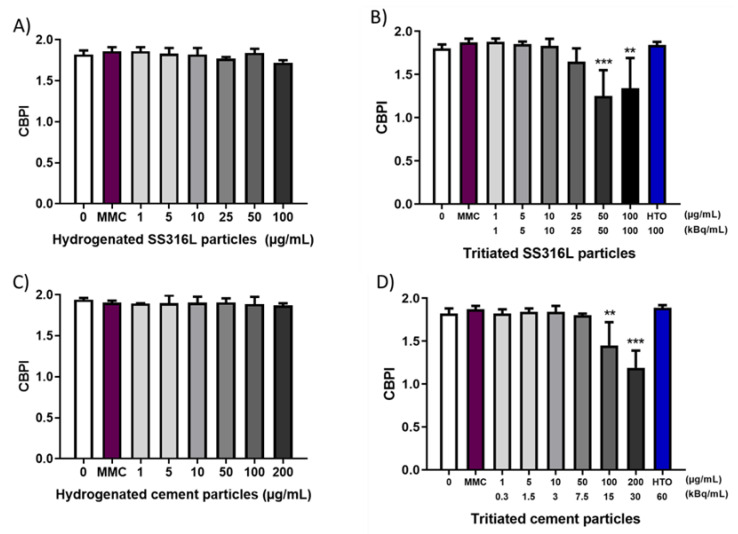
Cytokinesis Block Proliferation Index (CBPI) upon exposure to hydrogenated/tritiated SS316L (**A**,**B**) and cement (**C**,**D**) particles. Cells were also exposed to tritiated water with an activity corresponding to the highest activity of particles (100 kBq/mL and 60 kBq/mL). Each bar represents the mean ± SD of two independent experiments. Asterisks indicate statistically significant difference compared to untreated cells evaluated by one-way ANOVA, with *p*-value of *p* < 0.01 (**), or *p* < 0.001 (***).

After 24 h exposure of BEAS-2B cells to particles and controls (culture medium and HTO), the cytokinesis-block proliferation index (CBPI) was determined (Figure 5). No difference was observed comparing the CBPI of hydrogenated particles at all tested concentrations to untreated cells, indicating that stainless steel and cement particles were not cytostatic (Figure 5A,C). In contrast, tritiated particles impaired CBPI at the highest tested concentrations while HTO did not (Figure 5B,D).

Based on CBPI data, the effects of the exposure to particles on the cell proliferation rate were assessed by calculating the percentage of induced cytostasis (Table 1). Indeed, an inhibition of cell division could occur due to a toxic effect on various components necessary for cell division or as a result of the activation of cell cycle checkpoints following DNA or chromosome damage.

It is noteworthy that only tritiated stainless steel and cement particles induced a strong decrease in cytostasis at concentration above 50 and 100 µg/mL, respectively. HTO, at activity corresponding to the highest tested concentrations of tritiated particles, did not induce cytostasis.

Using CBMN assay in combination with centromere labeling, particles-induced chromosomal damages were also investigated and, among them, the discrimination between chromosome loss (leading to centromeric micronuclei; MN Crest +) and chromosome breakage (leading to acentromeric micronuclei; MN Crest −) was performed.

Mitomycin C (MMC) was used as a genotoxic positive control, causing a significant increase in the frequency of NM compared to the control. As a clastogenic agent, MMC induces chromosomal breakage leading to the formation of an acentromeric micronuclei (MN Crest −).

**Figure 6:**
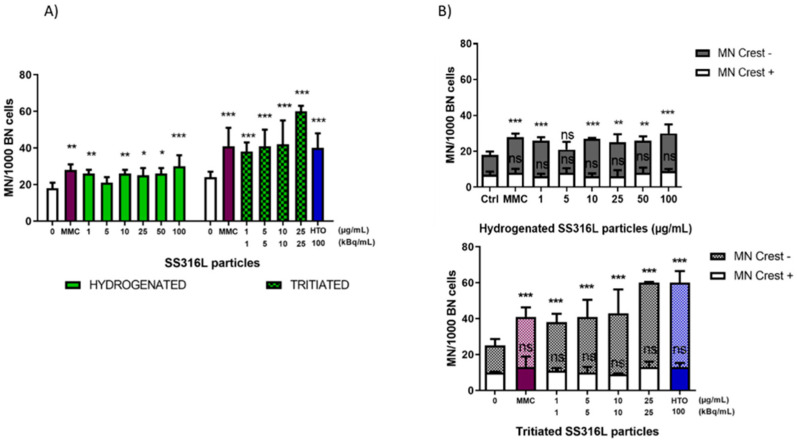

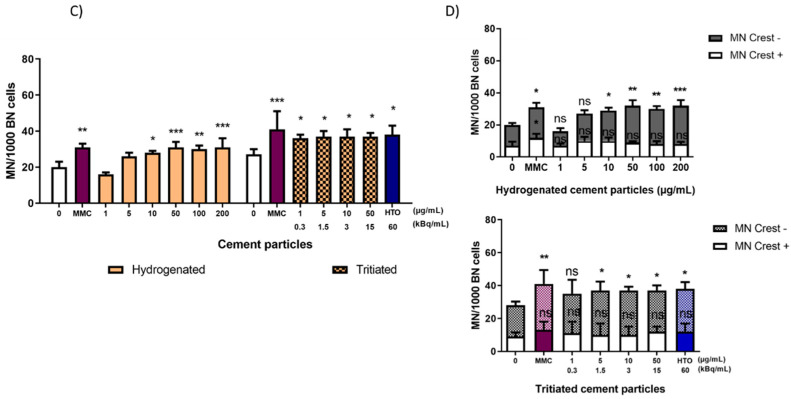
Micronuclei frequency in BEAS-2B cells exposed to SS316L and cement particles. MMC (0.1 µg/mL) was used as clastogenic positive control. Cells were also exposed to HTO with an activity corresponding to the highest activity of particles (100 kBq/mL and 60 kBq/mL). Centromere labeling (Crest) was performed to discriminate between MN issued by a whole chromosome loss (MN Crest +) and MN resulting from chromosome breakage caused by DSB (MN Crest −). Each bar represents the mean ± SD of two independent experiments. Asterisks indicate statistically significant increase compared to untreated cells determined by Chi-square, with *p*-value of *p* < 0.05 (*), *p* < 0.01, (**) or *p* < 0.001 (***).

As shown in Figure 6, a significant increase in MN frequency was detected, following exposure to hydrogenated and tritiated stainless steel and cement particles.

We can note that, following exposure to hydrogenated stainless steel particles, the increase in MN frequency was significant at 1 µg/mL and in the concentration range of [10–100] µg/mL, but no dose-related effect was clearly evidenced. Following exposure to the tritiated stainless steel particles, a clear dose-dependent and strong increase (*p* < 0.001) was evidenced in the concentration range of [1–25] µg/mL (Figure 6A).

Following hydrogenated cement particles exposure, a significant and dose-related increase in MN frequency was observed in the concentration range of [10–200] µg/mL, whereas tritiated cement particles induced a significant increase in MN frequency at lower concentrations in the range of [1–50] µg/mL (Figure 6C).

Exposure of cells to HTO also resulted in a significant increase in MN frequency.

Cement particles as well as SS316L particles induced mainly MN Crest −, suggesting that such particles exerted genotoxic effects via the formation of double strand breaks (DSBs) leading to chromosomal breakage (Figure 6B,D). HTO also displayed clastogenic potential (Figure 6B,D).

#### 2.1.6. Oxidative Stress

**Figure 7:**
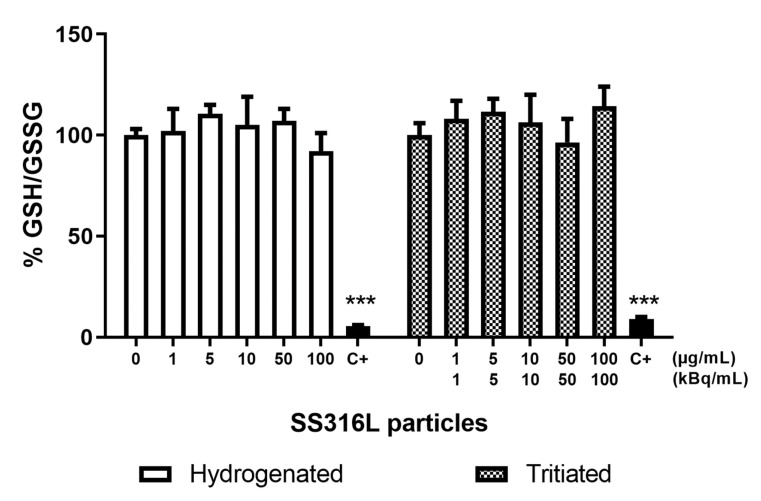
Oxidative stress evaluated by GSH/GSSG ratio in BEAS-2B cells, following SS316L particles exposure. Menadione (20 µM) was used as positive control (C+). Data are presented as mean ± SEM of two independent experiments, each in duplicate. Statistical significance was evaluated by one-way ANOVA followed by Dunnett’s multiple comparisons test, *** *p* < 0.001.

As oxidative stress is known to be one of the main pathways leading to DNA damage [30], the ability of particles to generate oxidative stress in cells was investigated. The ratio of reduced to oxidized glutathione was determined following particles exposure. As seen in Figure 7, none of the tested conditions induced an oxidative stress able to disturb the GSH/GSSG ratio except for the positive control consisting of 20 µM menadione. Following exposure to cement particles (tritiated and hydrogenated), no significant modification in the GSH/GSSG ratio was observed (data not shown).

### 2.2. Cytotoxicity of Tritiated Stainless Steel and Cement Particles in an In Vitro 3D Human Airway Epithelia Model

The MucilAir^TM^ human lung epithelium model was used in order to study and compare the toxicity of tritiated and non-tritiated particles on a three-dimensional cell model. Moreover, this 3D cell system allowed to quantify the transfer of tritium across the pulmonary epithelial barrier.

This model, which mimics the functioning of the human airway epithelium, has the potential to recapitulate toxicity responses through the respiratory function when exposed to a variety of pathogens or chemicals [26]. We recently used such a model to assess the toxicity of tritiated tungsten particles [31] and to quantify cytotoxicity and tritium absorption after administration of HTO to the tissue for 24 h, at different activity levels (up to 33 kBq µL^–1^ cm^–2^). A simple dosimetric model was developed to estimate the upper limits of the dose to cells, starting from the administered activity and considering water transport through the tissue [28].

#### 2.2.1. Epithelial Integrity and Metabolic Activity

TEER was measured upon exposure to particles and HTO at concentrations 165 and 550 kBq/mL (activity of tritiated cement and stainless steel respectively), at the end of exposure (day 1), then at day 4, 7, 10, and 14 post-treatment.

As seen in Table 2, the positive control (1% Triton-X 100) exerted a severe and permanent reduction of TEER. At the other tested conditions, TEER was not decreased at the end of exposure and until day 14. A small and transient increase of TEER was detected for the lowest activity level of HTO, at day 7.

Cell viability was also evaluated, based on metabolic activity measurement. Viability was not decreased by the exposure to tritiated particles or HTO, either at the end of the 24 h exposure, or 7 or 14 days afterwards, while the positive control exerted a drastic decrease (Table 3). Overall viability data confirm that culture conditions remained good over the entire investigated time frame.

#### 2.2.2. Transepithelial Passage of Tritium and Cellular Accumulation

To evaluate the absorption of tritium by the epithelial pulmonary MucilAir^TM^ tissue, the extracellular and intracellular amounts of tritium were quantified from day 1 to day 14 after exposure using liquid scintillation counting. The extracellular quantification was performed on mineralized apical and basolateral cell culture media.

At the end of exposure to tritiated cement and HTO, a low fraction of tritium (3% to 9% at day 1) remained in the apical media, while a high amount (67% to 72% at day 1) was able to translocate in the basolateral compartment (Table 4). The behavior of SS316L particles was different; the majority of tritium amount (54%) was present in the apical media at day 1.

From day 1 to day 14 post-exposure, the quantity of tritium still present in the apical and basolateral compartments severely and rapidly decreased up to less than 0.1% compared to day 1.

Finally, to quantify the intracellular level of tritium upon MucilAir^TM^ exposure, tritium quantification was performed on mineralized cells at day 14. Tissue cells appear to retain a negligible amount of the total activity, as less than 0.05% of tritium was found in cells regardless of the time point. Moreover, no further release of such internalized activity appeared to occur from cells to culture media.

## 3. Discussion

The toxicity of metal particles has already been studied for different metals that may contaminate at the work place [32,33,34]. Nevertheless, this work is the first one on the impact of accidental inhalation of tritiated steel and cement particles. In order to assess the hazard of particles that could be emitted during the dismantling of a nuclear power plant, the toxicity of tritiated cement and stainless steel particles was investigated using in vitro lung cell models. Hydrogenated particles were considered as the relevant controls to determine stress due to particles by themselves, which was then compared to both the particulate and radiative stress induced by tritiated particles.

During dismantling operations, the main route of exposure of workers to such particles is inhalation. Thus, we selected for the study in vitro human lung cell models. The human non-cancerous lung cell line BEAS-2B and the 3D in vitro model of human respiratory epithelium MucilAir^TM^ were chosen to study and compare the toxicity of hydrogenated and tritiated particles. These models are widely used to characterize the toxicity of different particles [35,36,37,38,39]. As a reference, BEAS-2B cells are among the most widely used immortalized cell lines for in vitro toxicology studies of agents for which the expected route of exposure is inhalation; this model, which resembles airway basal epithelial cells, offers the advantages of being easy to handle, not displaying inter-donor variability, and having an extended life span.

The MucilAir^TM^ model was used to determine the toxicity and especially the transfer of tritium across the pulmonary epithelial barrier. The MucilAir^TM^ tissue model allows kinetics to be monitored for several weeks to determine short- and long-term toxic effects and their reversibility. The two human lung cell models chosen are complementary since the BEAS-2B cell line is a proliferative test system, condition requested for determining a chromosome damaging effect, whereas Mucilair^TM^ is more representative of the human lung epithelium and enables the transepithelial passage of tritium.

Concerning the stainless steel particles, a review on stainless steel toxicity concluded that steel is likely to exert very low toxicity to humans [8]. For all the countless applications of stainless steel over many decades, harmful toxic effects have not been reported. In agreement with the literature [40,41], our results showed that an exposure of BEAS-2B cells to hydrogenated and tritiated stainless steel particles is associated with moderate to no cytotoxicity, assessed by measurements of the ATP content.

Among the huge diversity of toxicological endpoints, genotoxicity is of interest as the DNA and/or chromosome damage plays a critical role in pathological consequences of ionizing irradiation [15]. The alkaline version of the comet assay, measuring mainly DNA single strand breaks and alkaline labile sites, was used to assess DNA damage after exposure of cells to hydrogenated and tritiated particles. In our study, exposure to hydrogenated and tritiated stainless steel particles induced a similar increase in DNA damage. Using the same technique, a significantly higher amount of DNA damage was reported following 4 h exposure of A549 human lung cells to a concentration of 40 μg/cm^2^ (80 μg/mL) SS316L particles compared to the controls [40].

Hydrogenated and tritiated stainless steel particles induced chromosomal damage. Indeed, significant increases in the frequencies of micronuclei in BEAS-2B cells exposed to hydrogenated and tritiated stainless steel particles were observed. The chromosomal damage induction was dose-related and highly significant only for the tritiated particles. These observations suggest that a cumulative particulate and radiative stress could occur with tritiated particles. These micronuclei were predominantly negative to centromeric labeling, indicating that SS316L particles induced DNA double strand breaks (clastogenic potential).

Oxidative stress has been recognized as a key mechanism of particle-mediated toxicity, including lipid peroxidation and DNA damage. Indeed, DNA damage formation associated with the generation of oxidative stress was suggested by the significant increase of sites of formamidopyrimidine DNA glycosylase detected in A549 cells following exposure to micrometric stainless steel particles [42]. Moreover, stainless steel particles are composed of metals such as Fe, which can generate reactive oxygen species (ROS) by Fenton-type reactions.

The ability of particles to induce oxidative stress was thus assessed by quantifying cellular oxidized and reduced glutathione. Reduced glutathione (GSH), the most abundant low molecular weight thiol compound in cells, plays critical roles in protecting cells from oxidative damage and from the toxicity of electrophilic xenobiotics, and in maintaining redox homeostasis. Thus, an alteration in the balance between the reduced and oxidized forms of glutathione (GSH/GSSG) reveals oxidative stress at the cellular level.

Our results did not show any modification of the GSH/GSSG balance. As ROS were not quantified, we cannot exclude a moderate induction of ROS that could lead to oxidative damage without altering the GSH/GSSG balance.

Concerning cement particles, it was demonstrated that respirable cement particles did not induce cytotoxicity as assessed by the lactate dehydrogenase (LDH) assay in human primary epithelial cells from oropharyngeal mucosa exposed to concentrations up to 200 μg/cm^2^ [43]. A non-cytotoxic effect of cement particles was also observed on the hamster ovary cell line CHO in vitro [44] as well as in alveolar macrophages [34]. In agreement with these studies, no cytotoxicity, assessed by the measurements of ATP content, was observed in our study after 2 h and 24 h of exposure of BEAS-2B cells to either hydrogenated or tritiated cement particle concentrations ranging from 0 to 200 µg/mL.

Although no cytotoxic effects of cement particles were noted, DNA appears to be a privileged target of the toxicity exerted by cement particles. A study including cement warehouse workers reported that continuous exposure to cement dust was associated with an increased frequency of nuclear aberrations, based on a micronucleus cytome assay performed on exfoliated buccal cells. The median exposure time to cement dust among the exposed group was 14 years [7]. An increase in micronuclei frequency was also found in lymphocytes sampled in construction workers with durations of exposure varying from 10 to 30 years [6].

In our study, in vitro exposure of BEAS-2B cells to hydrogenated and tritiated cement particles also led to genotoxicity. A similar increase in the percentage of tail DNA (comet assay) was seen in cells exposed to hydrogenated and tritiated cement particles compared to the control. With the effects of tritiated and hydrogenated particles being similar, the comet assay did not reveal additional DNA single strand breaks, if any, induced by the tritium carried or released by tritiated particles (8 kBq/mL at the highest tested concentration). However, the involvement of tritium in the induction of primary DNA lesions cannot be excluded, as HTO also induced a significant increase in DNA damage as previously reported [45,46]. Our results also showed that hydrogenated and tritiated cement particles induced chromosomal damage in cells as indicated by the significant increase in the micronuclei frequency, highlighted by the CBMN test. The presence of tritium seems to enhance the chromosome damaging effects of the particles because tritiated particles induced a significant increase in the frequency of micronuclei at lower concentrations than hydrogenated particles. These observations suggest that a cumulative particulate and radiative stress occurs with tritiated particles. As noted with stainless steel particles, these micronuclei were predominantly negative to centromeric labeling, suggesting that hydrogenated and tritiated cement particles induced double strand breaks (clastogenic potential), as well as HTO.

An exposure of 4 h to cement particles was not associated with oxidative stress in alveolar macrophages [34], but following 12 h of exposure to cement dust, oxidative stress was reported in alveolar macrophages as indicated by the increase of intracellular ROS production and intracellular glutathione reduction [47].

A slight increase of oxidative DNA damage (8-OHdG) was observed in fibroblasts exposed to soluble fraction of cement particles for 1 and 12 h compared to the control [48]. In our study, no significant increase in the GSH/GSSG ratio was noted.

Interestingly, a high level of ROS and an increase in the number of DNA double strand breaks were found in MCF-10 cells exposed to low doses of HTO [49]. Another study reported that DNA strand breaks induced by HTO in human umbilical vein endothelial cells, assessed by comet assay and γ-H2AX immunostaining, were associated with modulations of DNA repair that could be regulated by the c-myc gene expression via miR34a [46]. One hypothesis is that the genotoxic effects of tritiated particles could be enhanced via an inhibition of DNA repair systems.

Particles-induced cytotoxicity and its reversibility were assessed with the MucilAir^TM^ model using a 14-day kinetic mode to determine short-term toxic effects and their reversibility.

Our results showed that an exposure to a high concentration (50 µg/cm^2^) of tritiated stainless steel and cement particles did not result in any toxicity as indicated by TEER measurement and metabolic activity evaluation by resazurin assay. No long-term deleterious effects were observed as shown by the absence of difference between TEER and metabolic activity measured before exposure and 14 days post-exposure. The activities associated with tritiated stainless steel and cement particles were, respectively, 16.5 kBq/well and 3.75 kBq/well. These results are in agreement with data obtained by Baiocco et al. showing a lack of toxicity following exposure of the MucilAir^TM^ model to HTO at the activities up to 330 kBq/well [28]. The lack of toxicity could also be associated with the presence of a mucus layer on the surface of MucilAir^TM^, which reduced the interaction between the particles and cells, thus protecting them from severe damage.

The MucilAir^TM^ model was also used to study the transfer of tritium across the lung barrier. Following exposure to HTO and tritiated cement particles, around 70% of the tritium was found in the basolateral compartment (Table 4). These data agree with the amount of tritium released from cement particles in the culture media (Figure 1). On the contrary, in the case of exposure to tritiated stainless steel particles, the majority of tritium is present in the apical compartment probably attached to the particles (Table 4). This behavior is different from that observed in the study of released tritium into culture media (Figure 2). Very low activities were measured in cells 14 days after the end of exposure, suggesting no accumulation of tritium in cells.

Overall, our results indicate that tritiated stainless steel and cement particles have little effect on cell viability. However, DNA and chromosome appear to be a target of the adverse effects of these particles, cell exposure being associated with an increase in primary DNA damage as well as chromosomal breakage.

## 4. Materials and Methods

### 4.1. Tools and Assays Used for the Characterization of the Cyto-Genotoxicity of Particles on BEAS-2B Cells

#### 4.1.1. Cellular Model: BEAS-2B

The BEAS-2B cell line is an immortalized but non-tumorigenic human cell line established from normal human bronchial epithelium obtained from a healthy individual [50]. This cell line has been widely used to determine various chemical, biological, and particle agents with potential pulmonary toxicity [9,42,51]. Cells were obtained from the American Type Culture Collection (CRL#9609, LGC Standards Sarl, Molsheim, France). They were cultured in culture flasks precoated with LHC basal medium supplemented with BSA (0.01 mg/mL), human fibronectin (0.01 mg/mL), and collagen (0.03 mg/mL) (Thermo Fisher Scientific, Illkirch, France). They were maintained in LHC-9 serum-free medium (Thermo Fisher Scientific, Illkirch, France) and BEGM^TM^ Medium (Lonza; Basel, Switzerland) in a humidified atmosphere of 5% CO_2_ at 37 °C. Before confluence, passage was performed twice a week using trypsin (0.25%)-EDTA (2.6 mM) (Thermo Fisher Scientific, Illkirch, France).

#### 4.1.2. Cells Exposure to Particles

##### Tritiated Stainless Steel Particles

For logistical and safety reasons, it was not possible to collect particles from a dismantled nuclear power plant. Thus, stainless steel particles were produced at the laboratory, cutting 316L stainless steel pieces that represented a decommissioning process within a nuclear facility. Then, the obtained aerosols were characterized. Because the production rate of particles from cutting operations was too low, a commercial powder (FF216030 Goodfellow Cambridge Limited, Huntingdon, UK) with similar characteristics to the home-made stainless steel particles was used. The elemental composition of the stainless steel particles was 69% *w*/*w* Fe, 17% *w*/*w* Cr, 10% *w*/*w* Ni, and 2% *w*/*w* Mo [28]. Scanning electron microscopy showed that these particles are spheroidal with size diameters from 1 to 8 µm [52]. A tritium loading was performed on the particles. Briefly, the tritiation process consisted of two steps of reduction at 450 °C under H_2_ with high pressure for 2 h, followed by the exposure of particles to tritium gas at 450 °C for 2 h [53]. This method resulted in a specific radioactivity of 1MBq/mg. A degassing time was observed to eliminate the unbound tritium and improve the safety of the tritiated sample. To assess the involvement of tritium in the various cyto-genotoxic endpoints following exposure to tritiated particles, all the experiments were also performed using particles loaded with hydrogen under the same conditions.

##### Tritiated Cement Particles

To mimic particles emitted during decommissioning, cement particles were produced at the laboratory, cutting a plate of hydrated Portland cement (water/cement ratio = 0.3) with a disk grinder. Particles were characterized for aerodynamic diameter (via optical counting) and the elemental composition of 46.6% *w*/*w* Ca, 10.8% *w*/*w* Si, 2.7% *w*/*w* Al, and 1.7% *w*/*w* Ti was confirmed following an alkaline or acid digestion protocol [54]. Cement particles were exposed to tritium gas at room temperature and we obtained a first batch with a specific radioactivity of 0.4 MBq/mg and another one with a specific radioactivity of 60 MBq/mg [53]. To assess the involvement of tritium in the various cyto-genotoxic endpoints following exposure to tritiated particles, all the experiments were also performed using particles loaded with hydrogen under the same conditions.

##### Suspensions Preparation and Cell Exposure

Particle suspensions were prepared extemporaneously. Stainless steel particles were first suspended in saline solution (NaCl 0.9%, CaCl2 1.25 mM, and Hepes 10 mM, Sigma-Aldrich, St. Quentin Fallavier, France) at the concentration of 4 mg/mL. Cement particles were directly suspended in culture medium at the concentration of 0.2 mg/mL. In both cases, dilutions were performed with culture medium in order to reach the chosen concentrations [0–200 µg/mL] corresponding to activities of 0–200 kBq/mL for SS316L particle and activities of 0–8 kBq/mL for cement particle suspensions used for the performed cell viability and comet assays, with 0–60 kBq/mL for cement particle suspensions used for the micronucleus assay. The size distribution of the stainless steel (0.1 mg/mL) and cement (0.2 mg/mL) particles suspended in the culture medium was measured in 1 mL of sample using an optical particle counter (Flowcell FC200S+ HR, Occhio, Belgium). Short-term aggregation of the SS316L particles (0.5 mg/mL) in saline solution (under agitation) was measured by laser diffraction (Malvern Mastersizer 3000, Malvern Panalytical; Orsay, France) with size measurements taken every 5 s over 10 m. Cells were treated for 2 or 24 h. As a second control, cells were also exposed to tritiated water. The HTO activities used were equal to the highest activity of the particles in each assay.

#### 4.1.3. Quantification of Tritium and Elemental Release from Particles

To determine tritium release, tritiated stainless steel particles were suspended in cell culture medium (100 and 10 µg/mL) and incubated at 37 °C with agitation (50 rpm). Two aliquots were collected at several time points over a 24-hour period. One aliquot was used to determine the total amount of tritium. This sample was mineralized by acid digestion using a mixture of HCl and HNO_3_ (3 V/1 V) and incubated for 24 h at room temperature. The second aliquot was centrifuged (10 min, 1635 g) to separate the soluble and particulate fractions and to determine the amount of tritium remaining in the supernatant (tritium released from particles). In the case of cement suspensions, mineralization was performed by acid digestion using a mixture of HCl and HNO_3_ (3 V/1 V) and incubation for 48 h at room temperature. The soluble fraction was obtained using Amicon^®^ Ultra-0.5 10kDa centrifugal filter devices (Merck-Millipore, Fontenay Sous Bois, France) (centrifugation 14,000 g for 30 m). Tritium quantification was performed in the supernatant of stainless steel samples, and the filtrate of cement samples as well as the mineralized samples of cement and stainless steel suspensions by liquid scintillation counting. Then, the following formula was applied: % tritium release = (amount of tritium in soluble fraction/amount of total tritium) × 100.

The experiments were performed in triplicates and results are expressed as mean ± SD.

Chemical stability was also evaluated over a 24-hour time period by measuring the elemental release for suspensions of 0.1 mg/L and 0.2 mg/L hydrogenated stainless steel and cement particles prepared in the culture medium, respectively. The suspensions for chemical analysis were prepared using the same filtration, centrifugation, and/or acid digestion procedures as noted above Analysis was performed using a PerkinElmer NexION 300X quadrupole inductively coupled plasma–mass spectrometer (ICP-MS, Villebon sur Yvette, France), with Fe, Cr, and Ni analyzed for the stainless steel particles and Ca and Al analyzed for the cement particles.

#### 4.1.4. Confocal Microscopy

Cells were seeded at 35,000 cells per well in 2-well chamber slides (Nunc™ Lab-Tek™ Chambered Coverglass™ System, Thermo Scientific, Illkirch, France). At 24 h post-seeding, a particle suspension (1 mL per well, final concentration 50 µg/mL) was added to each well. At the end of the 24-hour exposure, plasma membrane staining was performed by incubating the cells with fresh medium supplemented with wheat germ agglutinin-Alexa 488 at the concentration of 5 µg/mL (Biotium, Fremont, CA, USA) for 10 minutes at 37 °C. Then, the wells were washed twice with PBS and fixed using 4 % (*v*/*v*) paraformaldehyde in PBS (Electron Microscopy Sciences, Hatfield, USA). After washing twice with PBS, cells were permeabilized with 0.05% Triton-X 100 solution. Cytoskeleton staining and then nucleus staining were performed by incubating cells successively with phalloidin-tetramethylrhodamine B isothiocyanate (Sigma Aldrich Chimie Sarl; St. Quentin Fallavier, France) and DAPI. Finally, slides were mounted using ProLong^®^ Gold antifade reagent (Fisher Scientific; Illkirch, France) and stored at 4 °C. Slides were brought to room temperature prior to image collection on a Zeiss LSM 510 Meta confocal microscope (Carl Zeiss S.A.S.; Marly Le Roi, France). Images were taken at 400× magnification and light reflection (543 nm laser) was used to visualize the particles. The images were processed using Image J software.

#### 4.1.5. Cell Viability Assay: Intracellular ATP Quantification

The viability of cells was evaluated using CellTiter-Glo^®^ Luminescence Cell Viability Assay according to the manufacturer’s protocol (Promega; Charbonnières-les-Bains, France). This in vitro assay is based on an enzymatic reaction leading the conversion of luciferin, in the presence of ATP and Mg^2+^ into a luminescent compound, the oxyluciferin. The emitted luminescence was proportional to the amount of ATP, reflecting the cells metabolic activity. At 24 h after seeding, cells were exposed to the particles for 2 or 24 h. Luminescence signals were recorded using either a GloMax^®^ Explorer Multimode Microplate Reader (Promega; Charbonnières-les-Bains, France) or a Spectra Max M5 Microplate Reader (Molecular Devices; San Jose, CA, USA). For each experimental point, three independent assays were performed, each of them in triplicate (*n* = 9). The percentage of cellular viability was normalized to the unexposed control cells.

#### 4.1.6. Comet Assay

To detect the primary DNA damage induced by particles in BEAS-2B cells, the alkaline comet assay was performed as previously described [9]. BEAS-2B cells, seeded onto pre-coated 12-well plates (BD Falcon; Le Pont de Claix, France), were exposed to particles for 2 and 24 h. Then, after washing, cells were trypsinized. The cell pellet was resuspended in low melting point agarose, and then spotted onto glass slides treated with successive coatings composed by 1.6% and 0.8% normal melting point agarose, respectively. Cells were then lysed, and the DNA denatured in a MilliQ water solution containing NaOH 300 mM and EDTA 1 mM. After electrophoresis (25 V and 300–315 mA), samples were neutralized and dehydrated. Air-dried slides were stained with propidium iodide (PI) before imaging and data acquisition. For a negative control, cells were incubated only with culture medium, while slides treated with hydrogen peroxide (110 µM) for 5 minutes were used as positive controls. For each experimental condition, slides were prepared in duplicate. Samples were analyzed using either a fluorescence Axio Imager A2 microscope (Carl Zeiss S.A.S; Marly Le Roi, France) or a BX60 microscope (Olympus; Rungis, France) at 400X magnification. Data processing was performed using the Komet 6.0 software (Andor Bioimaging, Nottingham, UK). Results were expressed as mean % tail DNA ± SEM.

#### 4.1.7. Cytokinesis-Block Micronucleus Assay

To identify chromosome breakage and chromosome loss following particles exposure, the cytokinesis-block micronucleus assay (CBMN) in combination with centromere labeling was performed as previously described [9] and in compliance with the OECD487 guideline [20]. Briefly, BEAS-2B cells were seeded onto a four-well chamber slide system (Lab-Tek™ II Nalgene Nunc International, Villebon sur Yvette, France) and treated with increasing concentrations of particles for 24 h. Then, cells were washed and 3 µg/mL cytochalasin B (Sigma Aldrich Chimie Sarl; St. Quentin Fallavier, France) was added to the cultures to block cytokinesis. After 28 h of incubation, cells were fixed with 4% PFA. Mitomycin C (0.1 µg/mL) served as a positive control, whereas culture medium served as the negative one. Upon permeabilization, the cytoskeleton was stained with phalloidin-TRITC, while nuclei were stained with DAPI. Centromere labeling was performed successively incubating cells with Crest serum and Alexa 488 anti-human antibody. Finally, slides were mounted using ProLong^®^ Gold antifade reagent (Fisher Scientific; Illkirch, France).

CBMN was performed in duplicate, and slides were scored using either a fluorescence Axio Imager A2 microscope (Carl Zeiss S.A.S; Marly Le Roi, France) or a BX60 microscope (Olympus; Rungis, France) at 600× magnification. Micronuclei were only assessed in binucleated cells that had completed one nuclear division following exposure to the test compounds. For each experimental condition, the number of binucleated micronucleated cells was scored in 1000 binucleated cells.

To determine cytostasis, the Cytokinesis Block Proliferation Index (CBPI) was calculated by scoring mononucleated, binucleated, and multinucleated cells in the first 500 living cells analyzed in each sample.

CBPI, which indicates the average number of cell divisions completed by the cells, was calculated as follows:

[(1 × number mononucleated) + (2 × number binucleated) + (3 × number multinucleated)]/(500 viable cells).

The percentage of cytostasis was calculated as recommended by the OECD 487 test guideline:

{100 − 100 × [(CBPI exposed cells-1)/(CBPI control cells − 1)]}.

#### 4.1.8. Oxidative Stress Measurement

Oxidative stress following exposure to the particles was evaluated using the GSH/GSSG-Glo Assay (Promega; Charbonnières-les-Bains, France) as previously described [9]. This assay allows the quantification of oxidized and reduced glutathione in cultured cells, through a luminescence-based system.

According to the manufacturer’s instructions, cells were cultured in a 96-well plate and then exposed for 30 min to the particles. After exposure, cells were lysed. Luciferin detection reagent was then added, and plates were further incubated for 15 min before luminescence was read on a GloMax^®^ Explorer Multimode Microplate Reader (Promega; Charbonnières-les-Bains, France). Data were analyzed by subtracting the GSSG reaction signal from the total glutathione to obtain the value of reduced glutathione in the sample. Then, the GSH/GSSG ratio was calculated using the following formula:

(Luminescence Total glutathione−Luminescence GSSG)/[Luminescence GSSG/2]

Data were expressed as % GSH/GSSG ratio (mean ± SD) related to the untreated cells.

### 4.2. Tools and Assays Used for the Characterization of the Toxicity of Particles on MucilAir™

#### 4.2.1. Epithelium Model: MucilAir™

The MucilAir™ (Epithelix Sarl, Geneva, Switzerland) is an in vitro 3D model of human airway epithelia. This model is characterized by a pseudostratified columnar epithelium presenting beating cilia and mucus production. These cells were isolated from the human nasal cavity of a pool of human non-smokers donors without respiratory pathologies. Signed informed consent and ethical approval were obtained by the supplier. The MucilAir™ model mimics the upper respiratory tract structure of the human lung, including basal, goblet, and ciliated cells. This lung model is composed of the three cell types: basal, ciliated, and goblet cells, seeded on Transwell inserts [31,55].

Cells were supplied by the manufacturer ready to use in 24-well Transwell inserts of 0.4 μm pore size. Cells were cultivated with MucilAir™ serum-free culture medium (Epithelix Sarl; Geneva, Switzerland) and maintained at air–liquid interface configuration under standard conditions in a CO_2_ incubator (37 °C and 5% CO_2_) for up to three weeks (one week before the exposure and two weeks after exposure). The basolateral cell culture medium was changed twice a week, while the apical side was washed once a week with a sterile saline solution (NaCl 0.9%, CaCl_2_ 1.25 mM, and Hepes 10 mM). Renewing the basolateral media preserves the tissue homeostasis, and washing the apical compartment removes mucus and surface dead cells. Cellular morphology was assessed using optical microscopy twice a week.

#### 4.2.2. Exposure Protocol

One week after delivery, the MucilAir™ tissues were exposed to 50 µg/cm^2^ (550 µg/mL) of particles for 24 h. Particle stock solutions were diluted in saline solution and 30 µL of the suspension was applied on the apical compartment. Basolateral medium was collected and replaced first at the end of the exposure period, then twice a week for 14 days. At the end of the treatment period, the apical side was washed and the mucus collected, then the procedure was repeated twice a week for two weeks after exposure.

Cells were also exposed to HTO at the concentrations of 165 and 550 kBq/mL (corresponding to the activities of tritiated cement and SS316L particles, respectively) as a control for studying tritium transfer through the epithelium. Triton-X 100 (1%) was used as a toxicity positive control and saline solution as a negative control. These controls were applied in the same manner (30 µL on the apical side).

#### 4.2.3. Epithelial Integrity (TEER Measurement)

In order to evaluate the integrity of the MucilAir^TM^ epithelium, transepithelial electric resistance (TEER) measurements were performed using the EVOM Epithelial Voltohmmeter (World Precision Instruments; Hertfordshire, UK) as previously described [28,31]. These measurements occurred after particle exposure and twice a week for 14 days. Before measurement, 200 µL of saline solution were added onto the apical surface. Then, this volume was immediately removed in order to keep cells at the air-liquid interface. To calculate the TEER value of each insert, the mean resistance of a cell-free Transwell filter was subtracted from the obtained values.

#### 4.2.4. Resazurin Assay

To measure the cellular metabolism, the resazurin assay was performed (Sigma-Aldrich; Saint-Quentin Fallavier, France). This test is based on the measurement of the fluorescent signal of resorufin produced by the reduction of resazurin by mitochondrial reductases. The MucilAir^TM^ inserts were transferred in a new 24-well plate containing 6 µM resazurin in saline solution [28,31]. Resazurin solution (200 µL) was also applied on the apical surface and the plate was incubated for 1 h at 37 °C and 5% CO_2_. Then, 100 µL of the apical solution was distributed in a 96-well plate for fluorescence measurement (excitation filter = 544 nm and emission filter = 590 nm). At the end of the experiment, the inserts were re-transferred in a new culture plate containing fresh MucilAir^TM^ culture medium (700 µL per well). The remaining apical solution was removed without unsettling the epithelium and cells were put back into the incubator. The measurements were performed on four inserts per condition.

#### 4.2.5. Evaluation of Tritiated Particle Transfer

To estimate the cellular uptake of tritium and the transfer through the respiratory epithelium, the MucilAir^TM^ tissues were exposed to 50 µg/cm^2^ (550 µg/mL) of particles for 24 h. Apical and basolateral media were collected after particle exposure twice a week during 14 days and stored at −20 °C. At the end of the experiment, cells were trypsinized and collected. Samples (apical and basolateral medium, cells) were mineralized and radioactivity was measured as described above.

### 4.3. Statistical Analysis

The cell viability, primary DNA damage, Cytokinesis Block Proliferation Index, and oxidative stress in BEAS-2B cells as well as epithelial integrity and cell viability of the MucilAir^TM^ were statistically analyzed by one-way ANOVA followed by Dunnett’s multiple comparisons test. Micronuclei frequency was analyzed by Chi-square test. Statistical analysis was performed using GraphPad Prism version 8.1.2 for Windows (GraphPad Software; San Diego, CA, USA).

## Figures and Tables

**Table 1:** Cytostasis upon exposure of BEAS-2B cells to hydrogenated/tritiated SS316L (**a**) and cement (**b**) particles. Each value represents the percentage of cytostasis expressed as mean ± SD of two independent experiments.

(a)		
[SS316L] µg/mL	Hydrogenated	Tritiated
	% Cytostasis (Mean ± sd)	
0	1.6 ± 1.7	0.8 ± 0.8
1	0.6 ± 1.0	0.0 ± 0.0
5	1.8 ± 3.1	0.0 ± 0.0
10	2.0 ± 2.7	3.3 ± 5.7
25	6.3 ± 3.5	19.1 ± 14.7
50	0.0 ± 0.0	69.2 ±35.6
100	12.1 ± 6.9	59.3 ± 41.7
MMC	0.4 ± 0.7	0.7 ± 1.6
HTO		0.8 ± 1.4
**(b)**		
**[Cement]** **µg/mL**	**Hydrogenated**	**Tritiated**
	**% Cytostasis (Mean ± sd)**	
0	0.5 ± 0.8	1.8 ± 2.7
1	2.9 ± 0.3	2.2 ± 3.9
5	6.3 ± 6.4	0.6 ± 1.1
10	4.7 ± 4.7	2.1 ± 3.7
50	3.7 ± 3.2	3.8 ± 3.4
100	6.5 ± 7.1	43.4 ± 34.0
200	7.3 ± 3.3	75.3 ± 25.6
MMC	3.8 ± 1.5	1.7 ± 2.8
HTO		0.0 ± 0.0

**Table 2:** Epithelial integrity of MucilAir^TM^ following particles and HTO exposure, evaluated by TEER measurement. The measurements were performed before (D0) and at the end of exposure (D1), and then at day 4, 7, 10, and 14 post-exposure. The results are expressed as a percentage of TEER normalized to control conditions (C −) at the same time point. Triton-X 100 (1%), was used as a positive control (C +). Each value represents the mean ± SD of four individual inserts. Statistical significance was determined using one-way ANOVA followed by Dunnett’s multiple comparisons test: *p* < 0.05 (*) or *p* < 0.001 (***).

TEER	D1	D4	D7	D10	D14
C−	100 ± 30.1	100 ± 5.5	100 ± 3.1	100 ± 2.6	100 ± 6.2
C+	2.5 ± 0.4 ***	1.8 ± 0.3 ***	1.2 ± 0.3 ***	0.7 ± 0.3 ***	2 ± 0.3 ***
Hydrogenated cement	98.3 ± 16.1	103.4 ± 3.5	83 ± 2.8	82.4 ± 17.2	85 ± 21.5
Tritiated cement	83.9 ± 11.9	87.6 ± 21.9	114.2 ± 12.2	85.6 ± 5.1	118.5 ± 3.8
HTO 165 kBq/mL	75.5 ± 19.5	121.2 ± 3.7	120.6 ± 4.2 *	97.7 ± 7.5	103.1 ± 14
Hydrogenated SS316L	106.4 ± 6.3	78.6 ± 3.2	77.6 ± 20.7	102.8 ± 7.5	74.6 ± 17.3
Tritiated SS316L	82.3 ± 20.6	75.6 ± 32.4	80.5 ± 11.4	81.8 ± 16	97 ± 14.3
HTO 550 kBq/mL	84.7 ± 42.4	94.4 ± 44.1	104.4 ± 16.4	105.3 ± 2.2	91.2 ± 19.4

**Table 3:** Metabolic activity of MucilAir^TM^ following particles and HTO exposure, evaluated by resazurin assay. Analyses were performed at the end of exposure (D1), 7 days (D7), and 14 days (D14) post-exposure. Data are expressed as the percentage of cell viability (mean ± SD of four individual inserts) normalized to control conditions (C−) at the same time point. Triton-X 100 (1%), was used as a positive control (C+). Statistical significance was determined using one-way ANOVA followed by Dunnett’s multiple comparisons test: *p* < 0.05 (*), *p* < 0.01 (**), or *p* < 0.001 (***).

RESAZURIN	D1	D7	D14
C−	100 ± 18.3	100 ± 5.0	100 ± 14.4
C+	3.6 ± 0.4 ***	0.4 ± 0.1 ***	0.1 ± 0.3 ***
Hydrogenated cement	92.4 ± 16.4	98.3 ± 4.7	82.4 ± 7.7 *
Tritiated cement	161.7 ± 26.3 *	92.7 ± 6.1	114.3 ± 2.1
HTO 165kBq/ml	128.2 ± 31.8	89.5 ± 5.6	117.2 ± 9.8 *
Hydrogenated SS316L	83.2 ± 17.3	104.9 ± 4.2	119.3 ± 3.3 *
Tritiated SS316L	153.9 ± 24.2	97.4 ± 10.2	122.5 ± 5.1 **
HTO 550kBq/ml	147.1 ± 44.3	98.3 ± 4.0	120.3 ± 2.4 *

**Table 4:** Transepithelial passage of tritium and cellular accumulation. Tritium content was measured in both apical and basal media of MucilAir^TM^, as well as in cells, using liquid scintillation counting after mineralization of the samples collected at the end of the 24 h exposure (day 1), and at day 4, 7, 10, and 14 post-exposure. The results are expressed as percentage of tritium in the exposure solution (mean ± SD of four samples). <Q.L means that the obtained value was below the quantification limit.

% Tritium	Compartment	D0	D1	D4	D7	D10	D14	D14 in Cells
HTO 165 kBq/mL	apical	<Q.L	3.56 ± 0.11	<Q.L	<Q.L	<Q.L	<Q.L	<Q.L
	basolateral	0.21 ± 0.03	71.73 ± 2.92	<Q.L	<Q.L	<Q.L	<Q.L	
Tritiated cement	apical	<Q.L	8.89 ± 1.19	<Q.L	<Q.L	<Q.L	<Q.L	0.73 ± 0.11
	basolateral	<Q.L	71.56 ± 3.68	<Q.L	<Q.L	<Q.L	<Q.L	
HTO 550 kBq/mL	apical	<Q.L	3.88 ± 0.41	<Q.L	<Q.L	<Q.L	<Q.L	<Q.L
	basolateral	0.06 ± 0	66.57 ± 2.33	0.07 ± 0.01	<Q.L	<Q.L	<Q.L	
Tritiated SS316L	apical	<Q.L	53.63 ± 5.19	<Q.L	<Q.L	<Q.L	<Q.L	1.29 ± 0.37
	basolateral	<Q.L	25.06 ± 0.95	<Q.L	<Q.L	<Q.L	<Q.L	

## Data Availability

Not applicable.

## Supplementary Materials

The following supporting information can be downloaded at: https://www.mdpi.com/article/10.3390/ijms231810398/s1.
