# Supplementary figures and images for: Cyto-Genotoxicity of Tritiated Stainless Steel and Cement Particles in Human Lung Cell Models
Source: Int J Mol Sci. 2022 Sep 8;23(18):10398. doi: 10.3390/ijms231810398 (PMC9499181; doi:10.3390/ijms231810398)

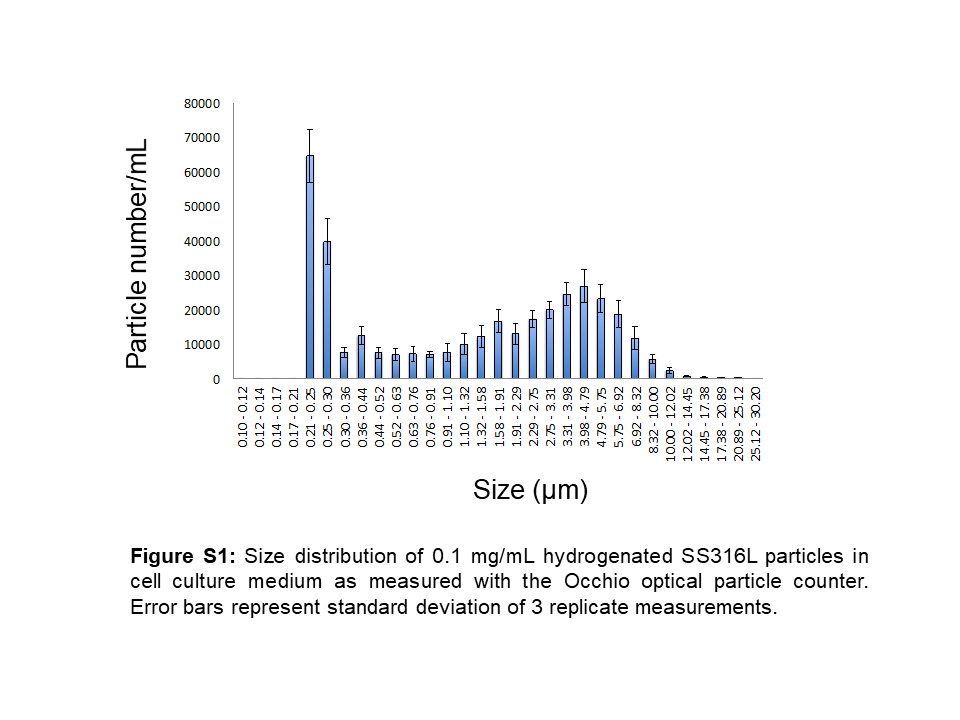

Supplement: Supplementary file 1 [file ijms-23-10398-s001.zip › Figure S1.TIF]

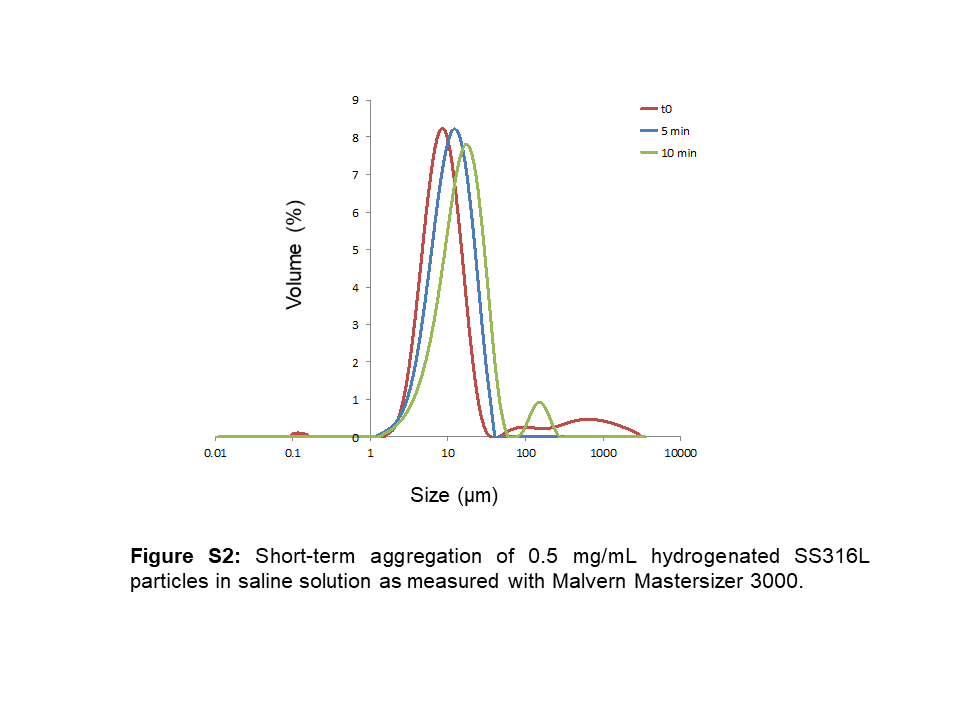

Supplement: Supplementary file 1 [file ijms-23-10398-s001.zip › Figure S2.TIF]

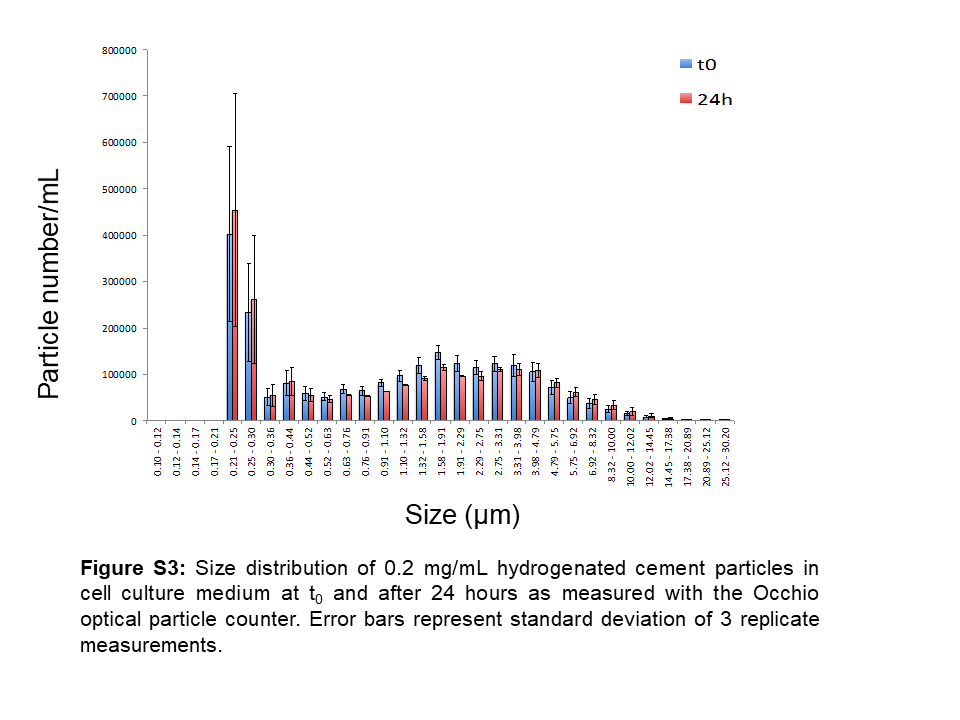

Supplement: Supplementary file 1 [file ijms-23-10398-s001.zip › Figure S3.TIF]
